# Exploring the Biopsychosocial Pathways Shared by Obstructive Sleep Apnea (OSA) and Central Serous Chorioretinopathy (CSC): A Literature Overview

**DOI:** 10.3390/jcm10071521

**Published:** 2021-04-06

**Authors:** Fabio Scarinci, Francesca Romana Patacchioli, Mariacristina Parravano

**Affiliations:** IRCCS-Fondazione Bietti, 00198 Rome, Italy; francesca.patacchioli@gmail.com (F.R.P.); mcparravano@gmail.com (M.P.)

**Keywords:** obstructive sleep apnea (OSA), acute/chronic central serous chorioretinopathy (CSC), psychopathology, stress, personality characteristics, functional diseases, biomedical/biopsychosocial illness model, choroid, comorbidity, quality of life

## Abstract

This study addressed the following question: “Is it possible to highlight the link between obstructive sleep apnea (OSA) and central serous chorioretinopathy (CSC) through common biopsychosocial pathogenetic pathways?”. The study was conducted through electronic searches of the PubMed, Web of Science, and Scopus databases. All relevant selected human research studies published from January 2003 to December 2020 were included. The scientific literature search was performed through repeated use of the words “OSA” and/or “acute/chronic CSC” paired with “biomedical/biopsychosocial illness model”, “psychopathology”, “stress”, “personality characteristics”, “functional diseases”, “comorbidity”, and “quality of life” in different combinations. Our literature search identified 213 reports, of which 54 articles were ultimately reviewed in this paper. Taken together, the results indicate that there is a cross-link between OSA and CSC that can be classified among biopsychological disorders in which various major biological variables integrate with psychological-functional and sociological variables; many of these variables appear in both diseases. This concept can have important implications for improving patients’ quality of life, thus providing the necessary strategies to cope with challenging life events even through nonpharmacological approaches.

## 1. Introduction

In the late 1970s, George Engel [1] published a landmark paper in Science proposing a biopsychosocial disease model [2,3], whose way of understanding the biopsychoneuroendocrine etiopathogenetic link between diseases seemed to be particularly applicable to obstructive sleep apnea (OSA) associated with central serous chorioretinopathy (CSC). The occurrence of OSA and CSC may be associated with various risk factors that can be briefly categorized into sociodemographic, biological, and psychosomatic-functional aspects, pointing out how these factors can contribute as a whole to illness and health [4].

The pathogenic role of OSA, characterized by repetitive upper airway occlusion episodes leading to apnea and excessive daytime sleepiness [5], is commonly associated with metabolic syndrome, including comorbid obesity and diabetes, as well as with cardiovascular and cerebrovascular diseases [6]. Moderate to severe OSAs are associated with a lower sleep spindle activity [7] and higher cortisol and inflammatory indices [8,9,10] when compared to healthy controls.

In addition, OSA has been associated with several ocular diseases [11,12] and has been considered an important risk factor for developing CSC [13]; OSA occurs most frequently in mid-life and more often in men than in women [14,15]. Exposure to increased levels of endogenous or exogenous glucocorticoids as well as psychological distress are among the various pathogenetic risk factors recognized for CSC [16,17,18].

OSA can be reversed quickly with a continuous positive airway pressure treatment [19]. Chronic CSC is best treated with photodynamic therapy, while a watch and wait approach is acceptable for acute cases [20].

Furthermore, psychiatric comorbidities have also been reported to adversely affect the quality of life of OSA- and CSC-affected subjects [21,22].

The purpose of this study was to provide a scientific literature overview to gather evidence underlying the possible association between OSA and CSC by an excursus on their sociodemographic, biological, and psychological-functional pathogenetic pathways.

This study could help broaden the knowledge on common pathogenetic mechanisms, and the findings could also help determine proper nonpharmacological therapies to keep patients’ quality of life as good as possible.

## 2. Methods

Eligible studies were original research articles involving human subjects published in peer-reviewed journals over the last 15 years until December 2020 and identified through searches of the PubMed, Web of Science, and Scopus electronic databases [23]. The search was performed through repeated use of words “obstructive sleep apnea (OSA)” and/or “acute/chronic central serous chorioretinopathy (CSC)” paired with “biomedical/biopsychosocial illness model”, “psychopathology”, “stress”, “personality characteristics”, “functional diseases”, “comorbidity”, and “quality of life” in different combinations.

**Figure 1:**
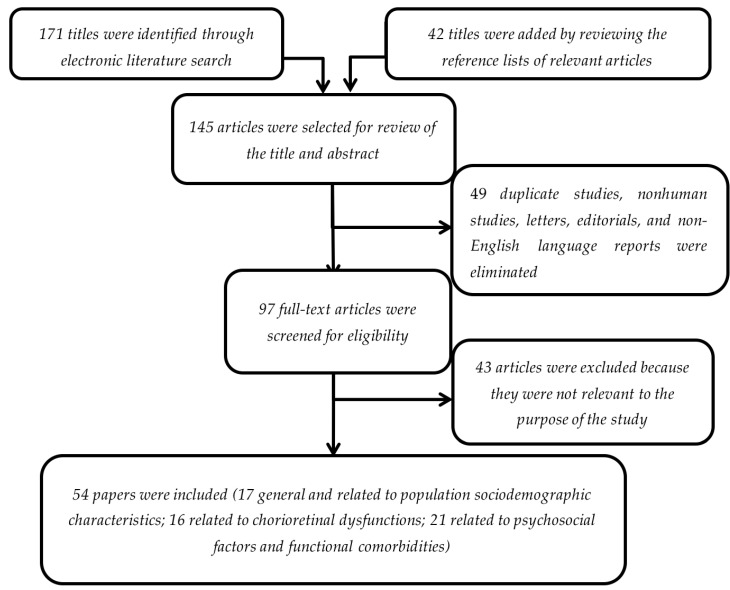
Literature selection method.

A flow chart describing the process of study identification is shown in Figure 1.

The initial search yielded 171 titles. In addition, 42 supplementary titles were included by scanning the reference lists of retrieved papers. All 145 abstracts were independently read by each coauthor; 49 reports (duplicate studies, nonhuman studies, letters, editorials, and non-English language) were excluded.

Of the remaining 97 abstracts, all full manuscripts were collected and independently reviewed by each coauthor for key information. Forty-three studies were excluded because they were not relevant to the purpose of the review.

All coauthors independently assessed eligibility by reviewing full-text articles; when it was unclear to one of the coauthors whether an article met eligibility criteria, the article was discussed among the research team and until all coauthors were in full agreement. As shown in the flow chart of the literature selection process (Figure 1), 54 key articles were ultimately identified to be reviewed in this paper.

## 3. Results and Discussion

The relationship between OSA and CSC has recently been further clarified in several studies reporting either a highly consistent [13,24] or less consistent magnitude [25] of the association between these diseases. Huon and coworkers [26] evaluated the incidence of OSA in a group of CSC subjects by estimating odds ratios with 95% confidence. Interestingly, the authors reported that the overall pooled odds ratio for OSA was 2.019 (*p* = 0.028) in CSC-affected patients, therefore concluding that screening for OSA should be considered in patients with CSC. More recently, Pan and coworkers [27] showed that older males might be particularly good candidates for OSA evaluation following a CSC diagnosis. Furthermore, it has been shown that due to the treatment of OSA, bilateral CSC is rapidly resolved [28].

In the following subsections, studies reporting possible biopsychosocial pathways shared between OSA and CSC are listed.

### 3.1. Sociodemographic Characteristics of OSA and CSC Populations

Male predominance has been described in OSA, although sex-specific pathogenetic mechanisms have been recently suggested to be associated with several important sociodemographic factors, including age, marital status, waist circumference, physical activity, hypertension, and sleepiness [29]. Similarly, it has been reported that the incidence of CSC was higher in men than in women [27]. However, being affected by OSA increases the risk of CSC equally in men and women [27].

### 3.2. OSA- and CSC-Related Chorioretinal Dysfunctions

Pertinent scientific literature addresses the issue of possible pathogenetic similarities between OSA-related and CSC-related chorioretinal dysfunctions [30,31,32]. OSA-affected subjects who have any additional disease show choroidal structural alterations that may have significance regarding the pathogenetic pathways of visual function alterations associated with OSA [33]. There are research studies in which a consistent association between choroidal structure and hypoxia-related vascular alterations and between choroidal structure and hypercapnia-related vascular alterations have been reported in OSA patients compared with controls. In several studies, optical coherence tomography was introduced as an effective tool for evaluating choroidal thickness alterations in OSA-affected patients, and it has been reported that choroidal thickness is reduced in patients with OSA [34,35,36]. More recently, it has been shown that subfoveal choroidal thickness is significantly reduced in 558 eyes of OSA patients compared with 226 normal controls [35].

In contrast, compared with the eyes of healthy subjects, it has also been reported that affected and fellow eyes of CSC patients have increased subfoveal choroidal thickness [37,38,39].

Patients with CSC who are also affected by moderate/severe OSA show decreased subfoveal choroidal thickness [13]. Interestingly, it has recently shown that choroidal thickness is significantly greater in patients with CSC and treated OSA than in patients with CSC and untreated OSA. Practically, CSC sufferers who have resolved OSA present increased choroid thickness as if they have CSC alone [40]. Further understanding how choroidal vessels are altered in CSC, and even considering how the relevant observation that nonvascular smooth muscle cells of the choroid may also play a role in the pathophysiology of CSC in response to an increased sympathetic tone, could help determine the pathogenesis of CSC [41].

Last, there are also studies showing no significant correlation between OSA severity and choroidal thickness in newly diagnosed subjects [42], and no significant change in choroidal thickness was found in patients with OSA in comparison with healthy controls, probably because of the small sample size evaluated [43]. With regards to the role of choroidal thickness in both diseases, further studies are warranted to explore the pathophysiology of choroidal thinning and its potential usefulness as a biomarker of systemic vascular dysfunction. Alongside these observations, it has been shown that in OSA-affected subjects, prolonged stimulation by high catecholamine levels might provoke focal retinal pigment epithelium (RPE) cell dysfunction, resulting in a breakdown of the outer blood-retinal barrier, followed by diffusion of fluids, promoting CSC [16,44,45].

### 3.3. Psychosocial Factors and Functional Comorbidities Associated with Both OSA and CSC

There have been traditional studies that have reported hypothalamic-pituitary-adrenal (HPA) axis and autonomic nervous system (ANS) dysregulations associated with both OSA and CSC, considered separately and as mutual comorbidities [14,42,43,44]. The similarities between OSA and CSC with regard to psychosocial distress have been the focus of numerous studies [16,28,46,47,48], and more recently, the identification of common stress-related etiopathogenetic processes has been reported [49].

Apneic events, both directly and indirectly, can lead to increased cortisol levels by disrupting the hormone regulatory response and activating the HPA axis [48].

Similarly, exposure to increased levels of endogenous or exogenous glucocorticoids is among the various etiopathogenetic conditions recognized for CSC [16,17,18]. Regardless, altered cortisol production is not the only pathophysiological link between OSA and CSC. In fact, OSA subjects have pathologically increased levels of circulating epinephrine and norepinephrine, both of which are risk factors for the onset of CSC [16,18,47,49,50].

Plasma concentrations of both catecholamines that are significantly correlated with macular edema are significantly higher in active CSC patients than in normal subjects and then decrease to normal in the remission phase of the disease [43].

Although OSA and CSC are to be classified as distinct pathologies, the HPA axis imbalance associated with elevated distress and mental health scores may even be attributed to similar psychosocial pathways shared by these diseases [18,51]. Psychosocial OSA-related disturbances may include difficulty concentrating, cognitive impairment, depression, and a general decrease in daytime quality of life [52,53]. However, it should be acknowledged that the association between OSA and depressive symptoms might also be influenced by uncontrolled confounding factors, including obesity, age, sex, and hypertension.

The relevant scientific literature generally reports that subjects affected by CSC have a significantly higher degree of psychological distress and even a “type-A” personality profile [54,55,56,57,58,59,60]. In particular, they revealed a higher tendency to present schizophrenia, hysteria, depression, psychopathic deviance, and hypochondriasis and exhibit higher levels of frustration and anticipatory anxiety than control groups [22,58,61].

On the other hand, similarities between OSA and CSC may also relate to clusters of unexplained symptoms, often grouped into syndromes, as is the case for fibromyalgia syndrome (FMS). FMS is a chronic painful condition typically referred to as a functional disorder. Functional disorders are largely considered psychosomatic [62], meaning that patients suffer from disease symptoms that are difficult to diagnose; similar complaints of sleep disturbances as well as significant rates of morning fatigue are reported for both FMS and OSA [63].

Furthermore, it has been reported that patients with FMS experience an increase in comorbid diseases, especially for those showing medium to high indications of depression [64].

Interestingly, it has been shown that FMS-related disorders may even be involved in the onset and especially the recurrence of CSC [65]. Reciprocally, the presence of an acute stress-anxiety condition often reported after a diagnosis of CSC may play a role in subjects being predisposed to developing FMS [65].

OSA itself constitutes an objectively stressful condition for the patient [50]; therefore, it could also be an independent risk factor responsible for the onset of induced CSC. As mere speculation, one may wonder whether OSA and CSC patients are truly “physically stressed” because of their pathology or whether their high score on the various psychometric scales represents a self-oriented belief and is thus a mere consequence of a higher physical reactivity in these individuals [53,55,56,57,58,59,60,66].

In summary, the clinical course of OSA and CSC can be attributed to similar psychosocial factors and lifestyles; in both pathologies, more marked psychological symptoms are associated with poorer quality of life. Subjects with vision impairments have twice the odds of reporting sleep disturbances independent of anxiety/depression and social isolation, two common problems affecting the quality of life in that population [67].

## 4. Conclusions

The current study was designed to explore, using a somewhat novel approach, whether OSA and CSC share a common biopsychosocial framework to gain a deeper understanding of their etiopathogenetic mechanisms. The results indeed suggest that OSA and CSC are both classifiable biopsychological disorders in which different major biological variables integrate with psychological-functional and sociological variables; many of these variables appear in both diseases.

In our opinion, the biopsychosocial approach to illness extends, but does not replace, the biomedical model and has the potential to provide the necessary strategies to cope with challenging life events even through nonpharmacological approaches. However, extending psychological traits into research ultimately contributes to improving a person’s quality of life by supporting their social and psychological needs.

## Data Availability

The data used to support the findings of this study are available from the corresponding author upon request.
